# Localized and Systemic Immune Response in Human Reproductive Tract

**DOI:** 10.3389/fcimb.2021.649893

**Published:** 2021-03-30

**Authors:** Rajendra Gudisa, Kapil Goyal, Parakriti Gupta, Mini P. Singh

**Affiliations:** Department of Virology, Postgraduate Institute of Medical Education and Research, Chandigarh, India

**Keywords:** reproductive tract, sexually transmitted infection (STI), virus, immunotherapeutics, immune system

## Abstract

Sexually transmitted infections (STIs) are one of the significant causes of morbidity and mortality among adolescents and adults across the globe and encompass all the infections transmitted *via* person-to-person sexual contact. In spite of the widespread approach being used, STIs remain under-reported and many infections have taken an epidemic turn. The biggest roadblock in this is the unraveled basis of immunopathology of these infections, hindering the discovery of potential targets for immunization. Thereby, it is of utmost significance to decipher the hidden basis of these STIs to control the increasing epidemic of less commonly studied STIs. A complex interplay between innate immune defenses, with resident microbiota and mucosal immune response serves as the basis of therapeutic approaches, by targeting the vital steps of this dynamic interaction. The characterization of pathogen-specific antibodies to significant immunogenic molecules may divulge the conceivable protective effects.

## Introduction

Sexually transmitted infections (STIs) are one of the significant causes of morbidity and mortality among adolescents and adults across the globe and encompass all the infections transmitted *via* person-to-person sexual contact. STIs account for one of the substantial public health problems, accounting for 333 million cases/year across the globe. STI is a broader term that encompasses a plethora of clinical syndromes that are acquired as well as transmitted through sexual activity. STIs are reported in ~25% of sexually active population and ~50% of all newly acquired ones. These account for a significant cause of mortality, morbidity, and daily-adjusted life years (DALYs) among young adult population, being second for young adult males and females. Bacterial STIs are more commonly encountered than viral and parasitic STIs. The viruses implicated in STIs include herpes simplex virus (HSV), human papillomavirus (HPV), hepatitis B virus (HBV), hepatitis-delta, hepatitis C, Ebstein-Barr virus, cytomegalovirus, molluscum contgiosum, human herpes virus-8, human papilloma virus. Among parasites, trichomonas and ectoparasites causing scabies and pediculosis are commonly reported. Of these, RNA viruses have grabbed principal attention; however, other pathogens are attaining a greater prominence lately, thereby, crowning into new classification of “first generation” and “second generation” infections. The global recognition of these STDs was acknowledged in 2000 by the United Nations after they included combatting STIs in millennium development goals. Risk factors and transmission dynamics in a community is multifactorial, the patterns being dependent upon the interplay between behavioral, cultural impacts, number of sexual partners, early coitrache, poor barrier protection, lack of awareness, and knowledge pertaining the transmission, societal, and economical factors. Although incidence and prevalence statistics is available, the existing data does not reflect the true prevalence in lack of the active screening of the same. World Health Organization has estimated 448 million cases caused by gonorrhea, syphilis, chlamydia, and trichomoniasis among 15 to 49 years of age. Concurrently, there are 33 million cases of HIV, and 24 million of HSV have also been reported. Moreover, HPV accounts for 70% of all cervical cancers and ~10% of women harbor HPV at any given point of time. Another matter of disquiet is the parent-to-child transmission of these STIs, commanding under-5 morbidity as well. To combat with the same, syndromic approach has been implemented in all the health care centers, to have an early diagnosis and management. However, in spite of the widespread approach being used, STIs remain under-reported, and many infections have taken an epidemic turn. The biggest roadblock in this is the unraveled basis of immunopathology of these infections, hindering the discovery of potential targets for immunization. Thereby, it is of utmost significance to decipher the hidden basis of these STIs to control the increasing epidemic of less commonly studied STIs. In this review, we delineate the updated dynamics of pathogen-associated molecular pattern with pattern recognition receptors, the interplay of antibody and cell-mediated immune responses to DNA viruses and parasites causing STIs.

## Immuno-Biology of Human Reproductive Tract

To decipher the immunological dynamics of STIs, an extensive understanding of biology of reproductive tract is mandatory since there is a gender variation in protective and immunological parameters. The immune protection is more complex in females, owing to the cyclic hormonal changes constantly occurring in the female genital tract to prepare the uterus for successful pregnancy. The female reproductive tract (FRT) is structurally dynamic in itself and has been divided into five anatomical zones accordingly; vagina, ectocervix, endocervix, uterus, and fallopian tubes, each of these having a distinct structural and immunological uniqueness. Earlier, upper reproductive tract was considered sterile as opposed to lower tract; however, this distinction has been stonewashed with time. It has been noted that Tc-99m–labeled microsphere suspensions, when placed in human vagina, traverse up to uterus within 10 to 20 min. The milieu of FRT is distinctively compartmentalized with temporal changes with regard to immune response. Vagina and ectocervix, the “gatekeepers for preventing the entry,” have increased chances of cross-contamination due to proximity to rectum; however, infection rate is comparatively low since it is inhabited by commensal flora and the immune cells (Quayle et al., 1998; Monin et al., 2020). Although mucosal immunity is the core player in this, but FRT mucosal immunity is quite distinct from other mucosal immune sites. Moreover, in addition to local mucosal immunity, systemic immune system has an equivalent role to play, as has been demonstrated in animal studies (Mestecky and Russell, 2000). Immune system is present throughout the FRT, and it is these immune cells that protect from external pathogens, commensal bacteria, and other threats and has been adapted in a way to facilitate the unique physiological functions like menstruation, pregnancy, and parturition, in addition to prevent these STIs. This immunological response to invading viruses and parasites is complemented by both innate as well as adaptive immune armors.

## Role of Commensal Flora

One of the major impelling factors that contribute in the local immunity of FRT is population of commensal flora, the microbiome. Human vaginal microbiome is characterized into five major groups, of which *Lactobacillus* predominates four and the other one is dominated by *Prevotella, Gardenella, Atopobium*, etc. Of all these, the role of *Lactobacillus* has been extensively studied and is known to protect individuals with microbiome dominated by strict aerobes other than *Gardenella* are at a higher risk of viral infections (Anahtar et al., 2015; Lewis et al., 2017; Ceccarani et al., 2019; Gupta et al., 2020).

## Role of Sex Hormones in Immunity

**Figure 1:**
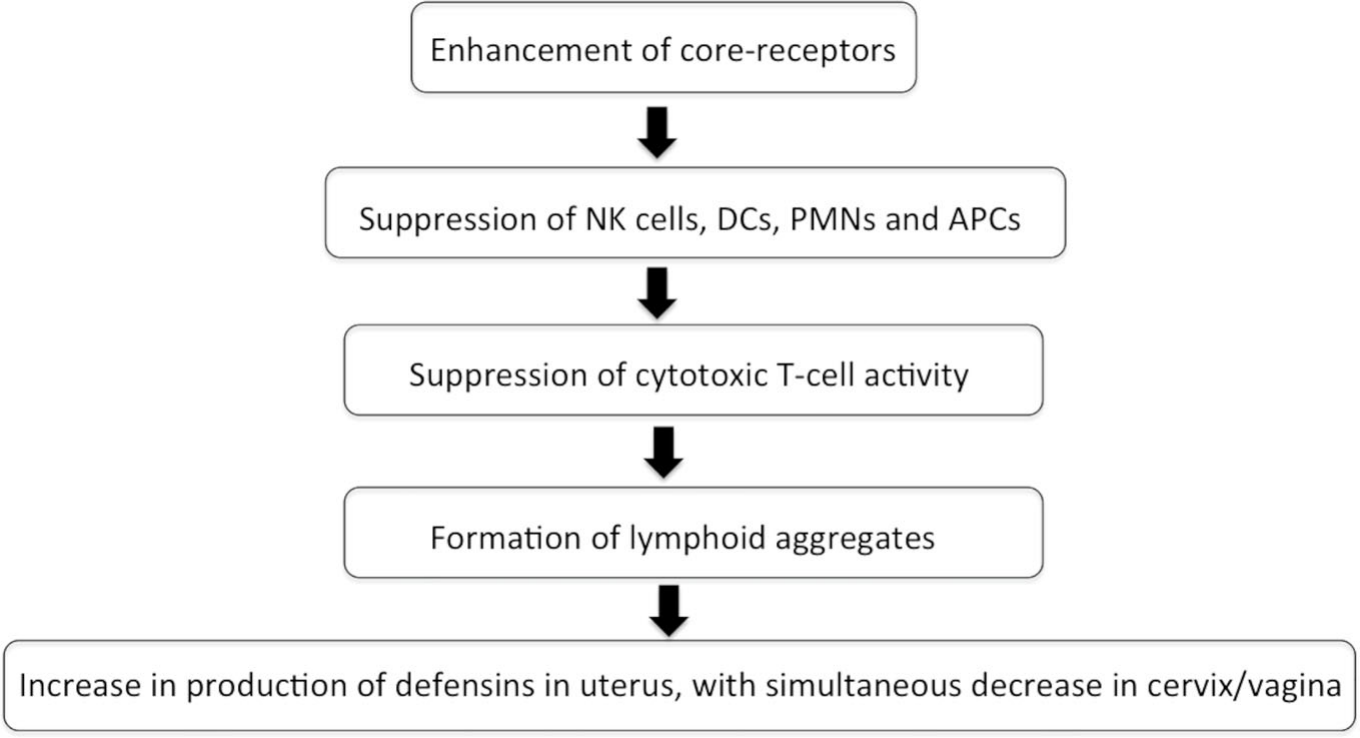
Flowchart depicting the effect of sex hormones during menstrual cycle on immune system in FRT.

Estrogen and progesterone act as the most instrumental stalwarts for modulating the immune reactions toward any foreign antigen. These hormones drive the immune reaction by influencing the migration of macrophages, dendritic cells, adhesion molecules, and chemotaxis, indirectly affecting the recruitment of varied cells of innate and adaptive armors (Fahey et al., 2005; Fahey et al., 2006; Wira et al., 2014). Estrogen and progesterone affect the FRT in a cyclical manner (**Figure 1**). Flow cytometry studies have revealed that CTL, lymphoid aggregates, and immunoglobulins decrease in number and function during the secretory phase of menstrual cycle, thereby, making it more vulnerable to infections (Givan et al., 1997; Mselle et al., 2007).

### Innate Immunity in FRT

**Figure 2:**
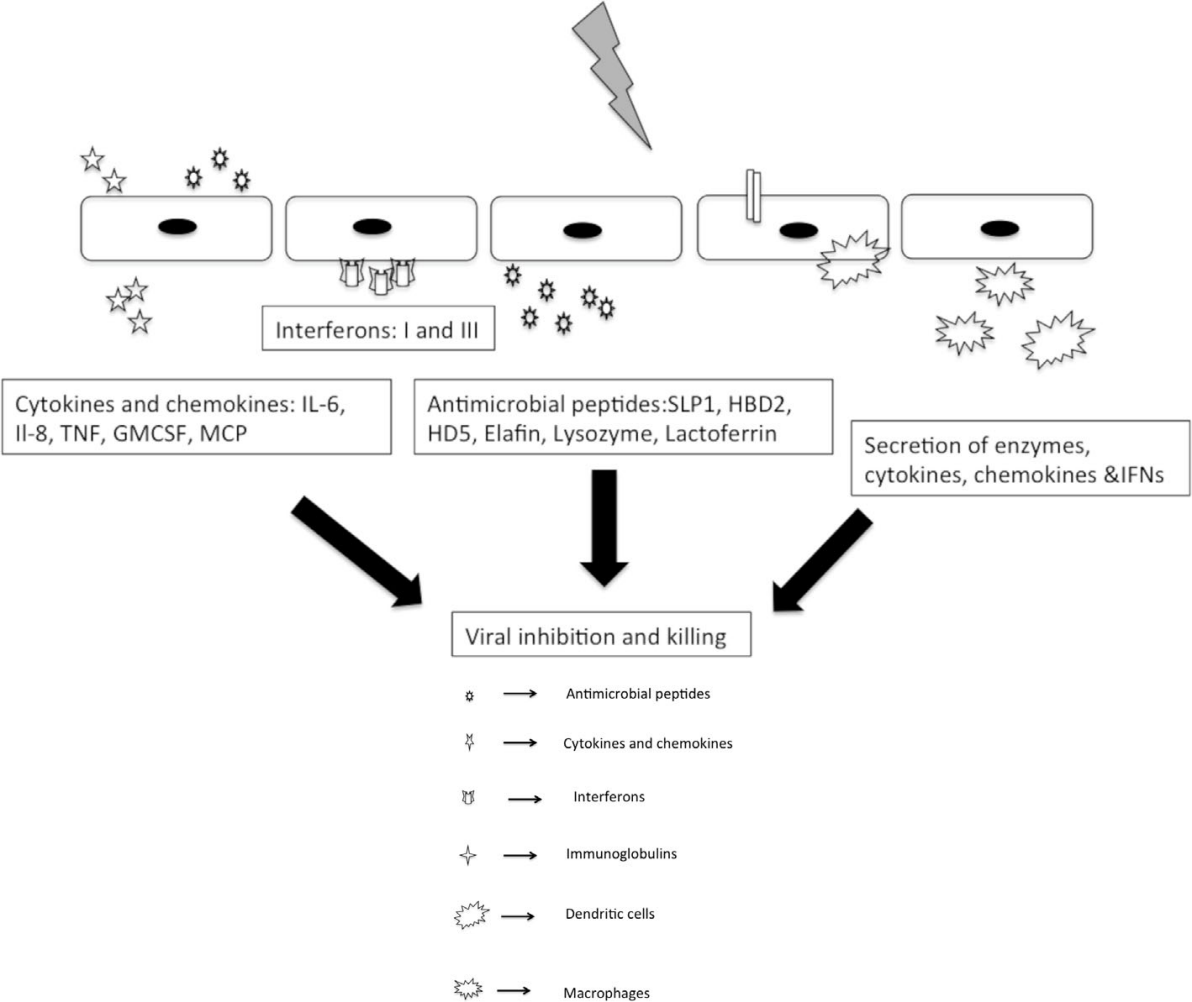
Innate responses to sexually transmitted viral pathogens.

Innate immunity forms the frontline barrier for all the viruses and parasites invading FRT. Innate response are more rapid and primitive than the adaptive response, resulting in a hackneyed inflammatory response and effectual pathogen elimination. The foremost sprinters of this system include prime interaction of pattern recognition receptors (PRRs) and pathogen associated molecular patterns and secretion of natural antimicrobial peptides (NAPs), with a dynamic population of immune cells present locally as well as periodically, which has the ability to migrate into uterus, cervix, and vagina. In addition to this, a dynamic balance of epithelial cells, fibroblasts, and immune cells is also a pre-requisite for triggering an immune response to a foreign antigen (**Figure 2**).

#### Epithelial Barrier

Epithelial cells form a first-line defense to combat any pathogenic intrusion. Epithelial cells have a role in bi-directional communication with immune cells to maintain a baseline effective level of protection that helps to differentiate the “self” and “non-self” antigens. Moreover, these cells have a pleiotropic capacity as sentinel sites. The barrier is composed mainly of non-keratinized stratified squamous cells that line vagina and ectocervix and columnar cells in uterus, with the presence of tight junctions between basal layers. These tight junctions are composed mainly of transmembrane proteins, occluding, claudin and junctional adhesion molecules (Grant and Wira, 2003; Wira and Fahey, 2004; Fahey et al., 2005). These serve as directional conduit; help maintain the integrity of FRT monolayers and aids epithelial cells to functionally polarize response to different stimuli. However, these are absent in vagina that permits the penetration of molecules to deeper layers, where “adherans junctions” are present to restrict the passage of escaped molecules. These epithelial cells on disruption by pathogenic attack lead to perturbation of cytoskeleton directly and release of cytokine-chemokines indirectly. Transforming growth factor (TGF-β) and tumor necrosis factor (TNF-α) are secreted by basolateral and apical surface, respectively. The secretion is mostly unwavering toward the luminal or apical compartment, leading to generation of a potent gradient for drawing immune cells toward the surface. An electrochemical gradient “transepithelial resistance” has also been prominently observed across the uterine monolayer, owing to the presence of tight junctions and barrier integrity. This transepithelial resistance is hormone-dependent, being the least in secretory phase (Simons and Wandinger-Ness, 1990; Tsukita et al., 2001; Grant and Wira, 2003; Shin et al., 2006). The same has been confirmed by administering selective estrogen receptor modulators (SERMs) that led to a decrease in viral infections by increasing the TER (Brotman et al., 2014). Another family of glycosylated proteins “murins” is also present in cervico-vaginal mucus. The pores of these proteins are large enough for the viruses to pass easily; however, they restrict the diffusion of penetrated particles by 800 folds. (Lai et al., 2010) Epithelial cells are also known to secrete a plethora of cytokines and chemokines, namely, HBP 1-14, HD5, elafin, SLP1, MIP-1 α, RANTES, SPF-1 α, and trappin-2, *via* upregulation of IL-1 β, IL-6, IL-8, and CCL-2 (Cocchi et al., 1995; Bleul et al., 1996; Quayle et al., 1998; Valore et al., 1998; King et al., 2003; Schaefer et al., 2005; Jv et al., 2008; Ghosh et al., 2009; Ghosh et al., 2010).

#### Fibroblasts

Fibroblasts are the active structural component that facilitate steroid hormone, playing a pivotal role in growth and development in epithelial membrane. They secrete hepatocyte growth factors, IL-18, RANTES, and MIP-3 α, which have a role to play in epithelial cell motility, proliferation, wound healing and embryogenesis (Kankuri et al., 2005; Skibinski et al., 2007; Shen et al., 2019).

#### Natural Antimicrobial Peptides

Natural Antimicrobial Peptides (NAPs) are whey acidic protein motifs containing peptides and are abundantly present at the epithelial surfaces and are activated once the membrane is disrupted by attack of pathogens. TLR 3, 7, 8, and 9 present at the mucosal surfaces recognize the PAMPs, in turn generating intracellular signals and cytokine-chemokine storm *via* activation of NF-kb pathway. NAPs are present throughout FRT and counter-act enzymes, preventing the host tissue damage. Secretory leucocyte protease inhibitor (SLPI) is one of the most potent NAPs, which inhibit mononuclear elastase, trypsin, and cathepsins. Elafin is another NAP and inhibits elastase and proteinase. NAPs are activated after pathogen attack (Helmig et al., 1995; Pfundt et al., 1996), during menstruation, secretory phase (King et al., 2003) and pregnancy (Hein et al., 2002; Fahey et al., 2005).

#### Defensins

Defensins are secreted by WBCs and epithelial cells after microbial attack. The most common defensins produced include β-defensins 1–4 and α-defensins. Defensins though present throughout the epithelium, have a unique temporal expression profile. Defensins 1–3 are more commonly present in uterine epithelium, 2 in cervico-vaginal epithelium, and 5 in fallopian tubes, vagina and cervix. Defensins 1, 3, and 5 are more abundantly produced in secretory phase, 2 in menstruation, and 4 in proliferative phase. (Quayle et al., 1998; Valore et al., 1998; King et al., 2003)

#### Toll-Like Receptors

Toll-like receptors (TLRs) are the most abundant group of PRRs present in FRT that play an indispensable role in recognition of conserved motifs of pathogenic antigens. FRT has all the 10 TLRs with a temporal profile. Upper FRT is rich in TLR-4, while TLR-2 is more common in fallopian tubes and cervix. Vaginal epithelial cells are lined with TLR 1, 2, 3, 5, and 6; however, endocervix is rich in TLR 1, 2, 3, and 6. TLR 1–9 is abundantly encountered in endometrial epithelial cell lines and TLR 2 and 4 in fallopian tubes and endometrium. (Krikun et al., 2007) Moreover, this TLR expression is cycle-dependent as well, but many studies have delineated contradictory results. Few studies have shown higher expression of TLR 2 and 4 in peri-menstrual phase, while secretory phase favors TLR 2-6, 9, and 10. Similar selective expression has been seen in pregnancy as well that favors the expression of TLR 2, 4, and NOD 1, 2 in trophoblasts. (Costello et al., 2007; Krikun et al., 2007)

### Immune Cells

#### Mononuclear Cells

Monocytes and macrophages circulate throughout FRT and mediate immune recognition as well as microbial elimination *via* phagocytosis. These cells also induce secretion of cytokines and chemokines, *via* down-regulation of HBP2, IL-8, and IL-1. Macrophages form 10% of all white blood cells (WBCs) of FRT. These are present in highest numbers in endometrial stroma and connective tissues, however, is cycle-dependent. On the contrary, vaginal macrophages remain constant in number, irrespective of the hormonal changes. These are distinct from gastrointestinal tract as these express more of CCR5 and CXCR4, making the sites more susceptible to infections.

#### Dendritic Cells

Dendritic cells are the primary armor chunks of antigen presentation. These can be either myeloid or plasmacytoid, based on embryonic origin. DCs are present in subepithelial stromal layer in endometrium an in epithelial layer in vagina. Of these, pDCs form the pillars of innate immune system of FRT owing to the sensitivity to TLR 7 and 9, subsequently, leading to secretion of interferons III and I. These professional APCS are pivotal in generation of adaptive immune response as well. DC-SIGN, a calcium-dependent carbohydrate binding protein, interacts with ICAM-2 on endothelial cells, inducing migration of DCs and triggering the immune response. (Schaefer et al., 2005)

#### NK Cells

NK cells account for 10% of systemic WBCs and 70% of mucosal WBCs, exhibiting phenotypic differences from other immune cells with presence of CD9, CD69, and CD94 markers. These are abundantly present in endocervix and endometrium, being absent from ectocervix. Upon attack by viruses and parasites, a pro-inflammatory cascade is triggered *via* generation of GM-CSF, IL-10, IL-8, and IFN-Υ. NK cells also generate angiogenic growth factors and leukemia inhibitory factors. (Wira and Fahey, 2004; Wira et al., 2005; Wira et al., 2013; Wira et al., 2014)

#### Polymorphonuclear Cells (PMNs)

Polymorphonuclear cells (PMNs) are present throughout FRT, with maximum population in fallopian tubes and progressively decrease toward the vagina. The PMN influx is preceded by IL-1 secretion. PMNs play a role in immune activation *via* secretion of elastase and activation of metalloproteases. Moreover, innate immune defense is activated more *via* disruption of epithelial barrier. The phagocytic and pathogen clearance is mediated by secretion of oxidative compounds, trappins, α-defensins, phospholipases, and cytokines (Wira et al., 2013; Wira et al., 2014).

#### Interferons

Interferons are the anti-viral compounds that modulate immune activity *via* autocrine and paracrine action. Three classes of IFNs have been noted, I, II and III. Type I interferons include 13, II include Υ and III include λ 1–3. Type I interferons play a role in innate and adaptive immune system, II in adaptive system, and III in innate system. A ubiquitous expression of IFNs has been reported throughout the FRT to induce an extensive antiviral state, creating a hostile environment for pathogen survival (Schaefer et al., 2005; Fahey et al., 2006; Brotman et al., 2014).

### Adaptive Immune System

**Figure 3:**
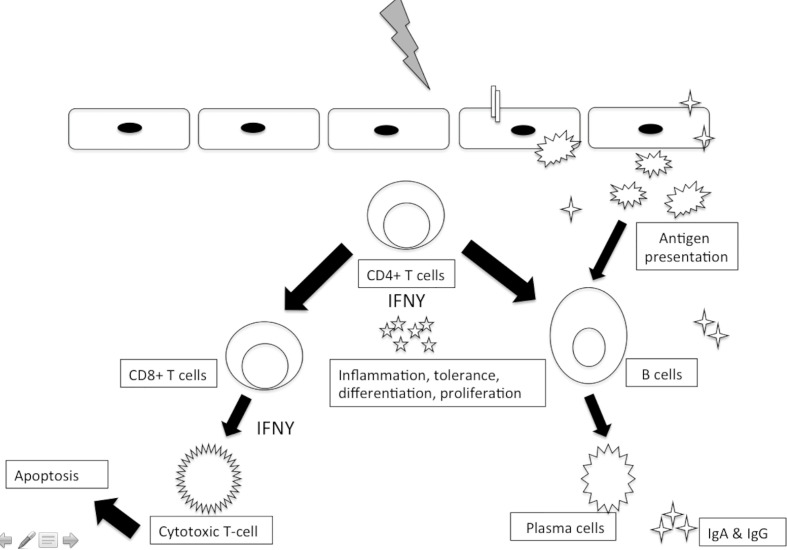
Adaptive immune responses to sexually transmitted viral pathogens.

Furthermore, complementing the innate immune activation, invading viruses and parasites also mount a more specific adaptive immune activation. This adaptive immune system has two arms, humoral and cell-mediated immunity (**Figure 3**).

#### Humoral Immune Response

Humoral immune system is distinct in secretion of immunoglobulins against the invading pathogens, which acts either *via* neutralization, opsonization or complement activation. Neutralization involves binding of antibody to free or bound antigens, preventing the cell entry. Opsonization, on the other hand, refers to coating of pathogen surface to facilitate the phagocytosis. Complement activation activates the complement pathway to induce an inflammatory response. Viral invasion leads to activation of B-cells, which in-turn interacts with CD4+T cells, culminating into differentiation of B-cells. These differentiated B cells play a role in generation of long-lived plasma memory cells (Charles A Janeway et al., 2001; Wira et al., 2005; Wira et al., 2013; Wira et al., 2014).

#### Immunoglobulins

Immunoglobulins are the major components of humoral immune system. A temporal shift has been noted, with IgG being more common in cervico-vaginal site, lower FRT. However, endocervical region is also quite rich in IgA. It has been revealed that IgG in FRT is plasma-derived, but IgA is locally generated. IgA produced is distinct in being resistant to IgA1 proteases and 70% of cervical IgA is locally produced. Moreover, transport of IgA is against the concentration gradient. An influence of hormones and contraceptives has also been demonstrated in FRT (Kutteh et al., 1996).

#### Cell-Mediated Immune Response

In addition to humoral immunity, cell-mediated immune (CMI) also plays a substantial role in FRT. T cells have been noted to be present in stroma of uterus, cervix and vagina. Moreover, distinctive intraepithelial lymphocytic clusters are also noted to be present within epithelial cells. Both the classes of T cells, CD4+ and CD8+ T cells are known to be present in FRT. Due to absence of mucosa-associated lymphoid tissue (MALT) in lower reproductive tract, the primary priming of CD4+ T cells occurs in draining lymphoid organs. However, upper tract has few traces of lymphoid associated tissues, which play a role in antigen presentation. CD4+T cells have a pivotal role in both humoral, as well as CMI immune arms, by helping CD8+T cells in their effective differentiation, mobilization, and concurrently, stimulating B cells to produce immunoglobulins. CD4+T cells might also take the role of cytotoxic cells to inhibit viral replication *via* secretion of IFN-Υ (Schaefer et al., 2005; Wira et al., 2013).

#### Cytotoxic T-cells

Cytotoxic T-cells (CTLs) are the main lytic cells of immune armor that exhibit the pathogen clearance *via* secretion of perforins and granzymes. Owing to the abundance of TGF-β and IL-16, pre-requisite for Th-17 differentiation, in genital tract, CD3+T cell-mediated cytolytic action is noted throughout the reproductive tract. The main inflammatory mediator cells in this regard are Th-17 cells, which trigger the immune response. Th-17 cells act as a double-edged sword, being of utmost significance in immune activation but simultaneously protecting the pathogen by inhibition of Th1 and Th2 response.

Another important subset of T cells, intraepithelial γδ cells are also present in genital tract. γδ T-cells rapidly initiate cytokine secretion and enhance the cytolytic activity. An important role has been reported in HSV infection. Moreover, these cells have a role to play in immunoregulation during pregnancy (Wira et al., 2013).

#### Memory Cells

Memory cells are present as a focus of cells within the submucosa and in intraepithelial sites. These are present as lymphoid aggregates, with a central B core, surrounded by CD4+T and CD8+T-cells, which is in turn encapsulated by macrophage shell. These cells can either be of CD4+, CD8+, or B-cell lineage and are unique to FRT, distinct from Peyer’s patches in intestines. The most substantial role is in rapid progressive response to secondary infections (Yeaman et al., 2001; Zhou et al., 2018).

### Immune Response in HSV

HSV-2 is a common STI that primarily affects the genital tract and, thereafter, spreads to the lumbosacral sensory ganglia to remain latent throughout the life. The innate response is attributed to NK cells and dendritic cells. (Morrison, 2002; Chew et al., 2009) NK cells are involved in cell recognition, cytokine production, and killing of infected cells. DCs are mainly involved in production of type I IFN. On the contrary, adaptive response comes into play pertaining progression of infection, latency, and limiting the spread of disease (Biron and Brossay, 2001; Lund et al., 2003). Cellular response has a substantial role in antiviral defense, with CD8+ T cells mounting the IFN production (Dobbs et al., 2005). CD4+ T cells have also been noted to provide protection in the infection, in absence of all other immune effectors (Iijima et al., 2008). Few studies have shown that mice deficient in CD+8 T cells were unable to clear the infection, while the same could be cleared rapidly in presence of ovalbumin-specific CD+8 T-cells. The same response was noted to escalate rapidly in presence of IFN-gamma, underlining the role of IFN-gamma in rapid clearance of HSV-2 infection, decreasing the viral titers at early stage of infection. Moreover, the study also highlighted the role of Fas and perforin-mediated cytolytic pathways required for the same (Dobbs et al., 2005).

### Immune Response in HPV

HPV is a ubiquitous viral agent with an infectious cycle bespoke to cell differentiation. HPV typically affects the basal keratinocytes after microabrasion; however, it expresses cascade of proteins only in upper layers of squamous epithelium, stratum spinosum, and stratum granulosum. Recent breakthrough studies have divulged that members of integrin family, a6b1 and a6b4, which are expressed on the basal cell surface, act as receptors for genital HPV infection (Gonçalves and Donadi, 2004). The virus amplifies from copy number of 1–10 to 50–100 episomes per cell, and this progression to nonregressing genital warts in few cases is attributed to lack of immune cells at the locally infected site. On the contrary, histological studies have revealed a huge infiltrate of CD4+ and CD8+ T cells in stroma and epithelium (Coleman et al., 1994). Innate response to HPV acts like the first line of defense with NK cells and macrophages, playing a substantial role and cytotoxic T-cells being the key layers in second line of defense (Scott et al., 2001; Sasagawa et al., 2012). There is generation of strong Th1 cell-mediated response in form of increase in milieu of pro-inflammatory cytokines with simultaneous upregulation of lymphocyte adhesion molecules. It has been noted that persistence of lesions and progression to cervical neoplastic lesions is more commonly associated with florid expression of viral genes and is frequently noted in immunosuppressed individuals unable to mount a strong immune response (Ho et al., 1998; Siegenbeek van Heukelom et al., 2017).

### Immune Response in CMV

CMV is one of the widespread herpesviruses causing persistent infections in humans, acquired in childhood or *via* sexual route. The primary infection is usually asymptomatic while the virus reactivates in immunodeficiency states. The virus remains latent in bone marrow in an episomal latency and can reactivate in response to varied inflammatory or stressful stimuli (Freeman et al., 2016). One such reactivation is noted in the form of increased shedding in genital tract, especially in PLHA. Extensive studies on host-virus dynamic interaction have revealed that CMV maintains a high level of specific T-cells engaged in struggle to confine the replication of virus to delimit the disease. CMV is known to directly up regulate the expression of pro-inflammatory cytokines, thereby escalating the inflammation. Furthermore, CMV has the ability to encode its own cytokines and receptor homologs, modulating the immune response, simultaneously, inhibiting HLA class I and II expression, thereby impairing the antigen presentation (Lisco et al., 2009; Söderberg-Nauclér, 2014).

### Distinctiveness of Male Genital Tract From FRT

Male reproductive tract (MRT) is the less understood and less complex, as compared to FRT. Owing to the differences in the ano-rectal proximity; STIs are less commonly encountered in males. The epithelium of MRT has temporal variation, with columnar epithelium present on the penile site, transitioning to non-keratinized stratified squamous epithelium at fossa navicularis to keratinized epithelium at the meatus. Apart from the epithelial lining, variation is also noted for mucin genes (MUC) genes, 1–20 with MUC 1 being more common at apical epithelium and MUC 4 throughout the tract. TLR expression also varies in MRT, TLR 9 being more expressed in uterine layers; 3, 8 in prostate and TLR 5, 6, and 8 in lamina propria. Antimicrobial peptides secreted in MRT include β-defensins, α-defensins, semenolysin, lysozyme, and protease inhibitors.

The variation in adaptive immunity includes the presence of macrophages in columnar epithelium in lamina propria. CD68+CD14+ T-cells are exclusively present in foreskin mucosa. Dendritic cells are abundantly located at fossa navicularis and meatus, with absence of dendritic cells in lower MRT. T-lymphocytes are known to be present throughout the MRT, with abundance of CD8+T-cells, which are positive for CD45RO. There is abundance of CD3+CD4+ T-cells in foreskin and submucosal epithelium, with CD3+HLADR+ phenotype.

### Role in Immunotherapeutics

The knowledge pertaining immune basis is not significant only to decipher the immunopathology of these STIs but also to delineate the role in prevention of these infections. One such role is highlighted in case of immunization, especially for HSV, HPV, trichomoniasis and CMV. Due to the inability to have a successful vaccine based on conservative approaches, it is mandatory to exploit the immunological aspects and targets of these infections so as to have a successful vaccination in place. The typical example is prophylactic HPV vaccines, which utilizes viral L1 protein assembled into virus-like (VLP) particles. These VLPs mimic the surface of actual virus to our immune system and generate an immune response by stimulating the production of antibodies (Starnbach and Roan, 2008). However, these vaccines are costly, require special preservative techniques and are prophylactic in nature, thereby limiting their use in therapeutics. Moreover, many recent breakthroughs in immunological basis have revealed other targets like E6 and E7 to be better therapeutic targets owing to their role in uninhibited cell proliferation and transformation. Many varied approaches are being tested for these STIs viz. prophylactic vaccines that target to generate humoral immunity; therapeutic vaccines that develop CMI to delimit the spread of infection; combination of both and vector-based approaches (Deligeoroglou et al., 2013). Such vaccines can be either peptide-based, DNA or RNA-based, viral-vector based, dendritic cell-based, VLP or modified tumor-cell based. These vaccines are currently under experimental stages for these STIs (Morrison, 2002; Cudmore and Garber, 2010; Anderholm et al., 2016). The insight of immunological basis of HPV infection has also paved the path for clinical trials using interferons and immune response modifiers. Imiquimod is one such example that has shown good response by generation of cytokines, simultaneously deescalating the viral replication (Tyring et al., 1998; Scott et al., 2001). Furthermore, the insight into immune basis of STIs has also led to proposition of “window of susceptibility” for these STIs and the same can be subjugated to make recommendations, preventive and vaccination strategies for these infections.

The inexorable incidence of these STIs paves the pathway for an urgent prerequisite to delineate the immunological basis to curb the progression of illness. A complex interplay between innate immune defenses, with resident microbiota and mucosal immune response serves as the basis of therapeutic approaches, by targeting the vital steps of this dynamic interaction. The characterization of pathogen-specific antibodies to significant immunogenic molecules may divulge the conceivable protective effects.

## Author Contributions

RG, PG, and KG contributed to the manuscript. MS conceptualized and corrected it. All authors contributed to the article and approved the submitted version.

## Conflict of Interest

The authors declare that the research was conducted in the absence of any commercial or financial relationships that could be construed as a potential conflict of interest.
